# Transcriptome analysis of auxin transcription factor OsARF17-mediated rice stripe mosaic virus response in rice

**DOI:** 10.3389/fmicb.2023.1131212

**Published:** 2023-03-09

**Authors:** Qiang Ma, Fengmin Wang, Weiqi Song, Chaorui Huang, Kaili Xie, Zhongyan Wei, Yanjun Li, Jianping Chen, Hehong Zhang, Zongtao Sun

**Affiliations:** State Key Laboratory for Managing Biotic and Chemical Threats to the Quality and Safety of Agro-products, Key Laboratory of Biotechnology in Plant Protection of Ministry of Agriculture and Zhejiang Province, Institute of Plant Virology, Ningbo University, Ningbo, China

**Keywords:** rice, rice stripe mosaic virus, transcriptome, auxin response factor (OsARF17), auxin signaling

## Abstract

**Introduction:**

Plant auxin response factors (ARFs) play an irreplaceable role in regulating the expression of auxin response genes. Our previous studies have indicated that auxin response factor OsARF17 plays a crucial role in plant defense against diverse rice viruses.

**Methods:**

Utilizing a comparative transcriptome analysis of Rice stripe mosaic virus (RSMV)-inoculated OsARF17 mutant rice plants, to further elucidate the molecular mechanism of OsARF17 in antiviral defense pathway.

**Results:**

KEGG enrichment analyses showed that the down-regulated differentially expressed genes (DEGs) belonged to plant-pathogen interaction and plant hormone signal transduction pathways were markedly enriched in *OsARF17* mutants under RSMV inoculation. Furthermore, Gene ontology (GO) analyses revealed that these genes were enriched in a variety of hormone biosynthetic process, including jasmonic acid (JA), auxin, and abscisic acid (ABA). RT-qPCR assays showed that the induction of plant defense-related genes, such as WRKY transcription factors, *OsAHT2* and *OsDR8*, and JA-related genes, were significantly suppressed in *OsARF17* mutants in response to RSMV.

**Discussion:**

Our study reveals that OsARF17-mediated antiviral immunity may be achieved through affecting the interaction between different phytohormones and regulating defense gene expression in rice. This study provides new insights into the molecular mechanisms of auxin signaling in the rice-virus interaction.

## Introduction

Rice (*Oryza sativa*) is one of the major grain crops in the world (Zeigler and Barclay, 2008; Muthayya et al., 2014). A number of plant viruses, including rice stripe virus (RSV), rice black stripe dwarf virus (RBSDV), rice yellow stunt nucleorhabdovirus (RYSV), rice stripe mosaic virus (RSMV), rice dwarf virus (RDV) and rice necrosis mosaic virus (RNMV) and so on, can infect and negatively impact the growth and development of rice, thereby endangering rice yield (Sasaya et al., 2014). Rice stripe mosaic virus (RSMV) is a negative sense of single stranded RNA virus in the genus *Cytorhabdovirus*, which was first detected in southern China in 2015 (Yang et al., 2016). Its genome encoding five structural proteins (nucleocapsid protein, phosphorylated protein, ribonucleic acid polymerase, glycoprotein and matrix protein) and two non-structural proteins (P3 and P6). Up to now, RSMV is the only *cytorhabdoviru*s to naturally infect rice, and the first to be transmitted by leafhoppers (*Recilia dorsalis*). The virus is widely distributed in southern China, with high infection rates in some areas. Rice infected with RSMV may exhibit symptoms such as slight plant dwarfing, increased tillering, yellow streaks on the leaves, twisted or crinkled mosaic patterns, delayed heading, cluster-shaped shortening of panicles, and mostly unfilled grains (Yang et al., 2016).

Plants produce small, multifunctional molecules called phytohormones, which are recognized by specific receptor proteins and trigger a signal transduction cascade. Auxin, the first discovered plant hormone, is an endogenous hormone containing an unsaturated aromatic ring and an acetic acid side chain, which plays an irreplaceable role in the growth and development of plants (Tiwari et al., 2001; Teale et al., 2006; Spaepen and Vanderleyden, 2011; Qi L. et al., 2012). The auxin regulating many processes depends on its concentration. Three key protein components involved in the auxin signal transduction pathway are: auxin receptor-associated SCF complex (SKP1), auxin protein with inhibitory function (Aux/IAA) and auxin response factor (ARF; Korasick et al., 2014; Weijers and Wagner, 2016). There are four main auxin signal transduction pathways: TIR1/AFBAux/IAA/− TPL-ARFs pathway, TMK1–IAA32/34—ARFs pathway, TMK1/ABP1-ROP2/6-PINs or RICs pathway and SKP2AE2FC/DPB pathway (Hagen, 2015). Transcriptional regulation of auxin signaling pathway depends on ARF, and ARF can bind to AuxRE, a cis-acting element of auxin response gene, to regulate the expression of related genes. Most ARFs contain three conserved functional domains, namely, a DNA-binding domain (DBD) at the N-terminal, a PBI domain for protein–protein interactions at the C-terminal, and an intermediate domain for transcriptional regulatory activity (Tatematsu et al., 2004).

Many studies have shown that the ARF transcription factor plays a key role not only in regulating plant growth and development, but also in defense against pathogen invasion (Liscum and Reed, 2002). In *Arabidopsis thaliana*, in the process of auxin-induced lateral root development, PRH1 is directly transcriptively regulated by ARF7 and LBDs, participating in the regulation pathway of ARF7-LBD, and then PRH1 regulates the occurrence of lateral roots by influencing the expression of *EXPANSIN* gene (Zhang F. et al., 2020). In wheat, TaARF15-A1 delays leaf senescence by negatively regulating senescence promotion and positively regulating senescence retarding genes, including genes related to plant hormone biosynthesis and metabolism as well as transcription factors (Li H. et al., 2022). In rice, after rice dwarf virus (RDV) infected rice, auxin content increased, and exogenous auxin promoted the degradation of OsIAA10 protein, which lifted the inhibition of OsIAA10 on the OsARF transcription factor bound by RDV and enhanced the resistance of rice to virus. OsIAA10 can interact with five OsARF transcription factors, among which OsARF12 and 16 positively regulate rice virus resistance, while OsARF11 negatively regulate rice virus resistance (Qin et al., 2020). Our previous studies have also shown that OsARF17, a transcriptional activator belonging to the ARF family, plays a defensive role in rice against various viral infections (Zhang H. et al., 2020). The RSMV inoculation experiment of *OsARF17* mutants showed that the mutants were more sensitive to the virus and had more severe symptoms compared to the control ZH11, indicating that OsARF17 is important for rice resistance to RSMV infection. However, the specific molecular mechanism of OsARF17 in antiviral defense pathway is still poorly understood. Therefore, we conducted RNA high-throughput sequencing experiments on RSMV-infected ZH11 and *OsARF17* mutant rice plants to investigate the role of OsARF17 in antiviral immunity in rice.

## Materials and methods

### Plant materials and growth conditions

The *OsARF17* mutant plants were obtained from background of wild type Zhonghua11 (ZH11; Zhang H. et al., 2020). Isolates of RSMV was kindly provided by Professor Guohui Zhou (South China Agricultural University, China). The seeds were geminated and grown into the greenhouse maintained at 28°C with a 14/10 h light/dark cycle.

### Insect vectors and virus inoculation assays

Inoculation of plants with RSMV using leafhoppers were performed as described previously (Zhang H. et al., 2020; Li L. et al., 2022). Briefly, for virus-free leafhoppers, insect adults were allowed to feed on each plant to lay eggs in glass beakers for 3 days, after which the leafhoppers were removed and detected by RT-PCR assays. Then, the plants fed by virus-free adults were further grown in the glasshouse for hatching to gain virus-free leafhoppers for subsequent experiments. To acquire RSMV, three-stage nymphs of the leafhoppers were fed on RSMV-infected rice plants for 3–4 days. Then, the nymphs were transferred onto rice seedlings for 10 days to allow the virus to circulate by the insects. RSMV-infected or virus-free leafhoppers were placed on rice seedlings (feed for 3–5 days) that had been sown in glass beakers for about 12 days (3–4 leaf stage). Then the infected rice seedlings were transplanted into the greenhouse, and the symptoms of the disease were observed during the growth of the rice seedlings. All rice materials were grown in a plant growth chamber at 28–30°C. About at 30 dpi, the rice seedlings showed the symptoms of yellow stripes and curled leaf tips. At this time, infected plant leaves were collected for RNA extraction and transcriptome analysis. The virus inoculation experiment was repeated three times with consistent results. And in each experiment, we used virus-free insects as negative controls.

### Total RNA extraction and RT-qPCR experiments

Total RNA was extracted from rice leaves by using the TRIzol reagent (Invitrogen, Carlsbad, CA, United States) according to the manufacturer’s protocol. By using HiScript III 1st strand cDNA Synthesis Kit (+ gDNA wiper; Vazyme, Nanjing, China) reverse transcribe 1–2 μg of total RNA into cDNA. The RT-qPCR analysis was performed using the ChamQ SYBR qPCR Master Mix (Without ROX) by the ABI7900HT Sequence Detection System (Applied Biosystems, Carlsbad, CA, United States). The 2^–ΔΔC(t)^ method was used to process the RNA expression level data. The rice reference gene OsUBQ5 (AK061988) was used to normalize the statistic. The RT-qPCR primers used in this study are listed in Supplementary Table S1. The samples were replicated three times, and the results were basically consistent.

### Analysis of transcriptome data

The mock and RSMV-infected rice samples of 30 dpi were collected, quick-frozen with liquid nitrogen, abraded into powder and RNA was extracted using the method described above. Three biological repeats and three to five leaves were collected from a different seedling for each biological repeat. Total RNA examination, library construction and sequencing were performed by LC Bio (Hangzhou, China) using the Illumina Hiseq 2000/2500 platform. The DEGs were identified using Cutadapt (Martin, 2011). The genes were considered with significance when log2 fold change ≥ 1, *p* ≤ 0.05 (Robinson et al., 2010). Functional analysis of the DEGs were performed using Gene Ontology (GO)^1^ and Kyoto Encyclopedia of Genes and Genomes^2^ pathways enrichment analyses tools.

## Statistical analysis

Statistical significance analysis, quantitative real-time PCR analysis were analyzed using one-way ANOVA with Tukey’s least significant difference tests. Each experiment was repeated at least three times, and data are represented as the mean. A *p*-value ≤ 0.05 was considered statistically significant, and asterisks indicate the statistical significance: ^*^ at the top of columns indicate significant differences, *p* ≤ 0.05. All analyses were performed using ORIGIN 8.0 software.

## Results

### OsARF17 Mutants displayed reduced resistance to RSMV

**Figure 1:**
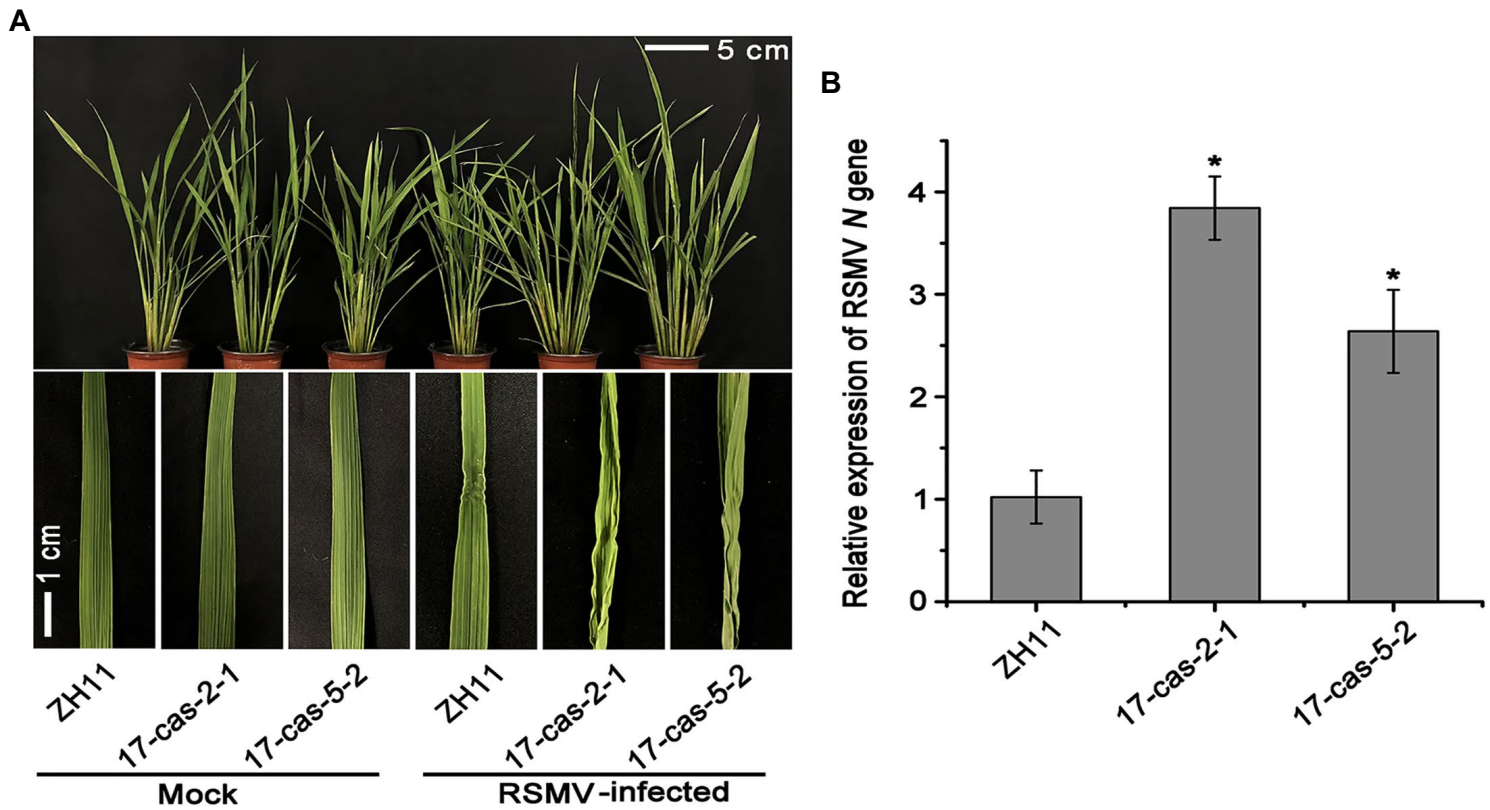
*OsARF17* mutants showed reduced resistance to rice stripe mosaic virus (RSMV) infection. **(A)** The phenotypes on RSMV-infected ZH11 and *OsARF17* mutant (*17cas-2-1* and *17cas-5-2*) plants. The mock-inoculated plants were symptom-free. The symptoms of diseased rice plants were observed and taken photos at 30 days after RSMV inoculation (Scale bars = 5 and 1 cm). Mock, healthy plants; RSMV-infected, RSMV-infected plants. **(B)** RT-qPCR analysis of the relative expression level of RSMV RNA *N* gene in RSMV infected ZH11 and *OsARF17* mutant plants. ^*^ at the top of columns indicate significant differences (*p* < 0.05) based on Fisher’s least significant difference tests.

We recently showed that the auxin transcription factor OsARF17 can enhance the resistance of rice to different types of virus infection (Zhang H. et al., 2020). In order to further clarify the mechanism of ARF17-mediated resistance to RSMV infection, the *OsARF17* mutant lines (*17cas-2-1* and *17cas-5-2*) were used to challenge with RSMV. The details of genomic location of OsARF17 mutations were showed in Supplementary Figure 1. RSMV infection exhibited distinct symptoms-plants slight dwarfing, the initial appearance of yellow stripes on leaves followed by mosaic and occasional twisting of some leaves. Our inoculation assays showed that RSMV-infected plants displayed marked curl of leaves and significantly increased tillering in plants. The inward-curled symptoms were more severe in *OsARF17* mutants than in ZH11 controls (Figure 1A). Then we measured the relative expression of RSMV *N* gene by RT-qPCR analysis, the results showed that RSMV *N* gene was dramatically more in two *OsARF17* mutants than in the control ZH11 plants (Figure 1B). This proved that the *OsARF17* mutants were more sensitive to RSMV infection, and consistent with our previous findings. Together, these data suggest that OsARF17 plays an inevitable role in rice defense against RSMV infection.

### Differentially expressed genes (DEGs) of *OsARF17* mutants and ZH11 plants In response To RSMV

To obtain a comprehensive view of the transcriptome changes of rice in response to RSMV infection, high-throughput RNA sequencing (RNA-seq) experiments were selected for the transcriptome analysis of *17cas-2-1* mutant line and ZH11 plants in response to RSMV infection. The two-week-old rice plants were inoculated with RSMV through viruliferous leafhoppers feed on rice seedlings for 3 days. About 30 days after inoculation, the rice leaves from mock and RSMV-infected plants were collected with three biological replicates for RNA-seq. Quality control and mapping information were listed in Supplementary Table S2. The differentially expressed genes (DEGs) from the 12 cDNA libraries were analyzed. Each library has more than 36 million valid Reads. The value of Q20% sequencing quality was above 99%. The value of Q30% sequencing quality was 96–97%. The GC content ranged from 51 to 53%. These results showed that the sequencing quality was good for further analysis.

**Figure 2:**
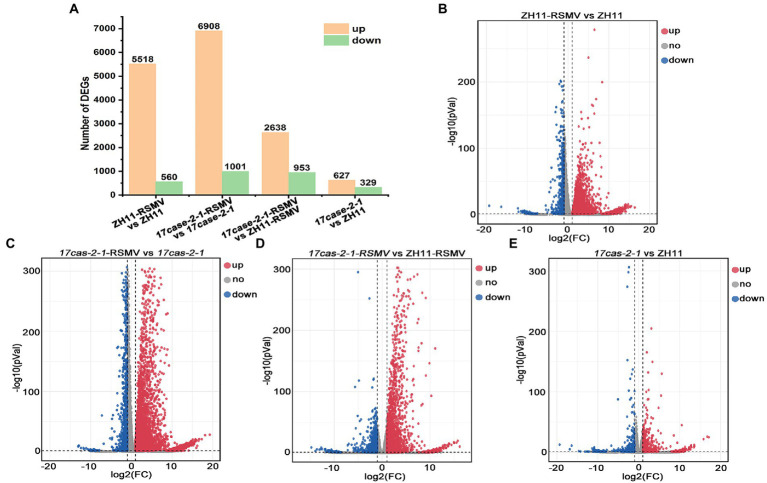
Differentially expressed genes (DEGs) of the rice in response to RSMV analysis. **(A)** Number of differentially expressed genes (DEGs) in ZH11–RSMV vs. ZH11 (RSMV-infected ZH11 plants compared to ZH11 control plants); *17cas-2-1*-RSMV vs. *17cas-2-1* (RSMV-infected *17cas-2-1* plants compared to *17cas-2-1* plants); *17cas-2-1*-RSMV vs. ZH11-RSMV (RSMV-infected *17cas-2-1* plants compared to RSMV-infected ZH11 plants); *17cas-2-1* vs. ZH11(*17cas-2-1* plants compared to ZH11 control plants). **(B)** The volcano map of differentially expressed genes in ZH11-RSMV vs. ZH11. **(C)** The volcano map of differentially expressed genes in *17cas-2-1*-RSMV vs. *17cas-2-1.* **(D)** The volcano map of differentially expressed genes in *17cas-2-1-RSMV* vs. ZH11-RSMV. **(E)** The volcano map of differentially expressed genes in *17cas-2-1* vs. ZH11. Red dots represent up-regulated significantly differentially expressed genes, blue dots represent down-regulated significantly differentially expressed genes, and grey dots represent no significant differences, respectively.

According to transcriptome sequencing, we found that a huge amount of host DEGs at the transcriptional level after rice plants infected with RSMV. The number of genes significantly up-regulated and down-regulated in ZH11 after RSMV infection was 5,518 and 560 (ZH11-RSMV vs. ZH11), 6,908 and 1,001 (*17cas-2-1*-RSMV vs. *17cas-2-1*), respectively (Figures 2A–C). When both were inoculated with the virus, we identified 2,638 differentially up-regulated genes and 953 down-regulated genes in the *17cas-2-1* mutant relative to ZH11 plants (*17cas-2-1*-RSMV vs. ZH11-RSMV; Figures 2A,D). Compared with the group of ZH11-RSMV vs. ZH11(6,078 DEGs), the number of DEGs (7909) in the group of *17cas-2-1-RSMV* vs. *17cas-2-1* increased slightly. In addition, a total of 956 DEGs were identified in *17cas-2-1* mutant plants, including 627 up-regulated genes and 329 down-regulated genes (*17cas-2-1* vs. ZH11; Figures 2A,E). This implied that the loss of *OsARF17* gene in rice inhibited the differential expression of some genes after RSMV infection. Overall, the results indicated that both viral infection and the knockdown of *OsARF17* cause significant interference in the transcription levels of many genes in rice.

### Analysis of DEGs suppressed in *OsARF17* mutants under RSMV inoculation

**Figure 3:**
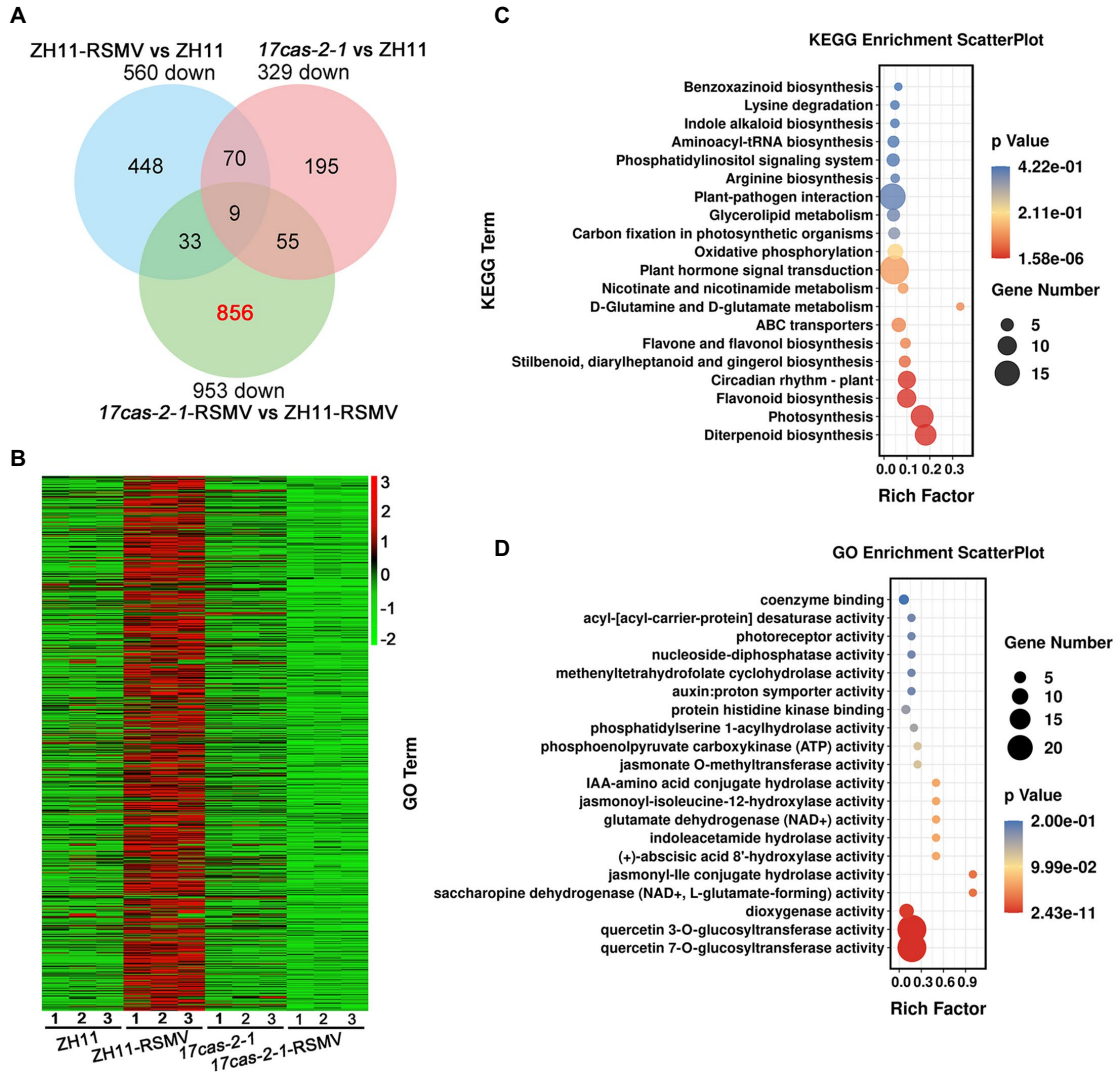
Analysis of the 856 of 953 differentially expressed genes (DEGs) of OsARF17-dependent genes were suppressed in *17cas-2-1*-RSMV vs. ZH11-RSMV. **(A)** Venn diagram illustrating the overlapping of down-regulated 856 DEGs in ZH11–RSMV vs. ZH11, *17cas-2-1*-RSMV vs. ZH11-RSMV and *17cas-2-1* vs. ZH11. **(B)** Hierarchical clustering of 856 DEGs in **(A)** based on the log2 fold change in transcript levels in ZH11 and *OsARF17* mutant plants under mock or RSMV inoculation. The hierarchical clustering analysis was performed on the FPKM values of all tested genes. **(C)** KEGG pathway enrichment analyses of 856-down genes (labelled in red) in **(A)**. **(D)** Gene ontology (GO) enrichment analysis of 856-down genes (labelled in red) in **(A)**. “Rich factor” shows the ratio between the number of DEGs and the total genes in this pathway. All differentially expressed genes were selected using cut-off *p*-value < 0.05 and fold-change > 2 compared with controls. Data were collected from three biological replicates, each containing a pool of three plants.

A Venn diagram analysis showed that 9 overlapping DEGs in all three comparison groups of ZH11-RSMV vs. ZH11, *17cas-2-1*-RSMV vs. ZH11-RSMV and *17cas-2-1* vs. ZH11, suggesting that these genes have been consistently differentiated in expression. We found that 856 genes out of the 953 down-regulated genes (about 90%) were specifically down-regulated in *17cas-2-1* plants after RSMV infection (Figure 3A). Interestingly, based on hierarchical cluster analysis the expression levels of these genes were obviously induced in RSMV-infected ZH11 plants (Figure 3B), indicating that these genes may be regulated by OsARF17. We then functionally analyzed the 856 DEGs about their Kyoto Encyclopedia of Genes and Genomes (KEGG) pathways to study the biological relationship of these genes. The candidate genes were first mapped to KEGG pathway terms. Hypergeometric tests are then applied to look for pathways that are significantly enriched in candidate gene profiles, relative to the overall genomic background. These pathways were enrichment in diverse metabolic pathways mainly including plant-pathogen interaction, plant hormone signal transduction, circadian rhythm-plant, diterpenoid biosynthesis, flavonoid biosynthesis and photosynthesis (Figure 3C). Notably, the two pathway terms, “Plant-pathogen interaction” and “plant hormone signal transduction,” were markedly enriched (Figure 3C).

Furthermore, the DEGs were classified according to Gene Ontology (GO) enrichment analysis. 856 down-regulated genes were highly enriched in a variety of enzyme activity (GO:0080044, quercetin 7-O-glucosyltransferase activity; GO:0080043, quercetin 3-O-glucosyltransferase activity; GO:0051213, dioxygenase activity; GO:0047131, saccharopine dehydrogenase (NAD+, L-glutamate-forming) activity; GO:1990206, jasmonyl-Ile conjugate hydrolase activity; GO:0010295, (+)-abscisic acid 8′-hydroxylase activity; GO:0043864, indoleacetamide hydrolase activity; GO:0004352, glutamate dehydrogenase (NAD+) activity; GO:0052694, jasmonoyl-isoleucine-12-hydroxylase activity; GO:0010178, IAA-amino acid conjugate hydrolase activity; GO:0030795, jasmonate O-methyltransferase activity; GO:0004612, phosphoenolpyruvate carboxykinase (ATP) activity; GO:0052739, phosphatidylserine 1-acylhydrolase activity; GO:0043424, protein histidine kinase binding; GO:0004477, methenyltetrahydrofolate cyclohydrolase activity; GO:0017110, nucleoside-diphosphatase activity; GO:0045300, acyl-[acyl-carrier-protein] desaturase activity), auxin: proton symporter activity (GO:0009672) and photoreceptor activity (GO:0009881; Figure 3D). Of them, “hormone biosynthetic process” and “signal transduction” were significantly enriched in the GO analysis, including jasmonate, auxin, abscisic acid and gibberellin.

Since *OsARF17* mutants were found to be more sensitive to viral infection than ZH11, we speculated that defense-related genes may be more suppressed in *OsARF17* mutants than that in ZH11 under RSMV infection. Therefore, we further analyzed the transcriptome data, using the criteria: log2 FC (fold change) of ZH11-RSMV vs. ZH11 ≥ 1 and > log2 FC of *17cas-2-1*-RSMV vs. *17cas-2-1*. The analysis showed that genes related to plant-pathogen interaction and plant hormone signal transduction genes, including OsWRKY transcription factors of *OsWRKY64* (Os12g02450), *OsWRKY14* (Os01g53040), *OsWRKY89* (Os11g02520), *OsbZIP transcription factors* of *OsbZIP70* (Os09g10840), *OsbZIP62* (Os07g48660), gibberellin 20 oxidase 2 gene, and genes related to disease resistance protein of agmatine hydroxycinnamoyl transferase 2 *OsAHT2* (Os04g56910) and defense responsive gene 8 *OsDR8* (Os07g34570), were significantly increased in ZH11-RSMV vs. ZH11 compared with *17cas-2-1*-RSMV vs. *17cas-2-1* (Table 1). These results support the speculation that defense-related genes were more suppressed in *OsARF17* mutants under RSMV infection, which may be due to the disruption of auxin signaling affecting the expression of defense-related genes.

**Table 1:** Partial representative differentially expressed genes.

Gene	Annotation	Log2FC (infected/uninfected)	
		RNA-seq	
		ZH11	*17case-2-1*
LOC_Os01g53040	WRKY transcription factor 14	1.84	1.57
LOC_Os11g02520	WRKY transcription factor 89	3.74	1.19
LOC_Os12g02450	WRKY transcription factor 64	2.27	0.89
LOC_Os09g10840	bZIP transcription factor 70	2.21	0.32
LOC_Os07g48660	bZIP transcription factor 62	3.18	1.13
LOC_Os04g56910	Agmatine hydroxycinnamoyl transferase 2 (AHT2)	10.17	6.08
LOC_Os07g34570	defense responsive gene 8 (DR8)	1.21	−0.69
LOC_Os08g44590	gibberellin 20 oxidase 2	2.63	0.39

### Analysis of DEGs induced in ZH11 and suppressed in *OsARF17* mutants

**Figure 4:**
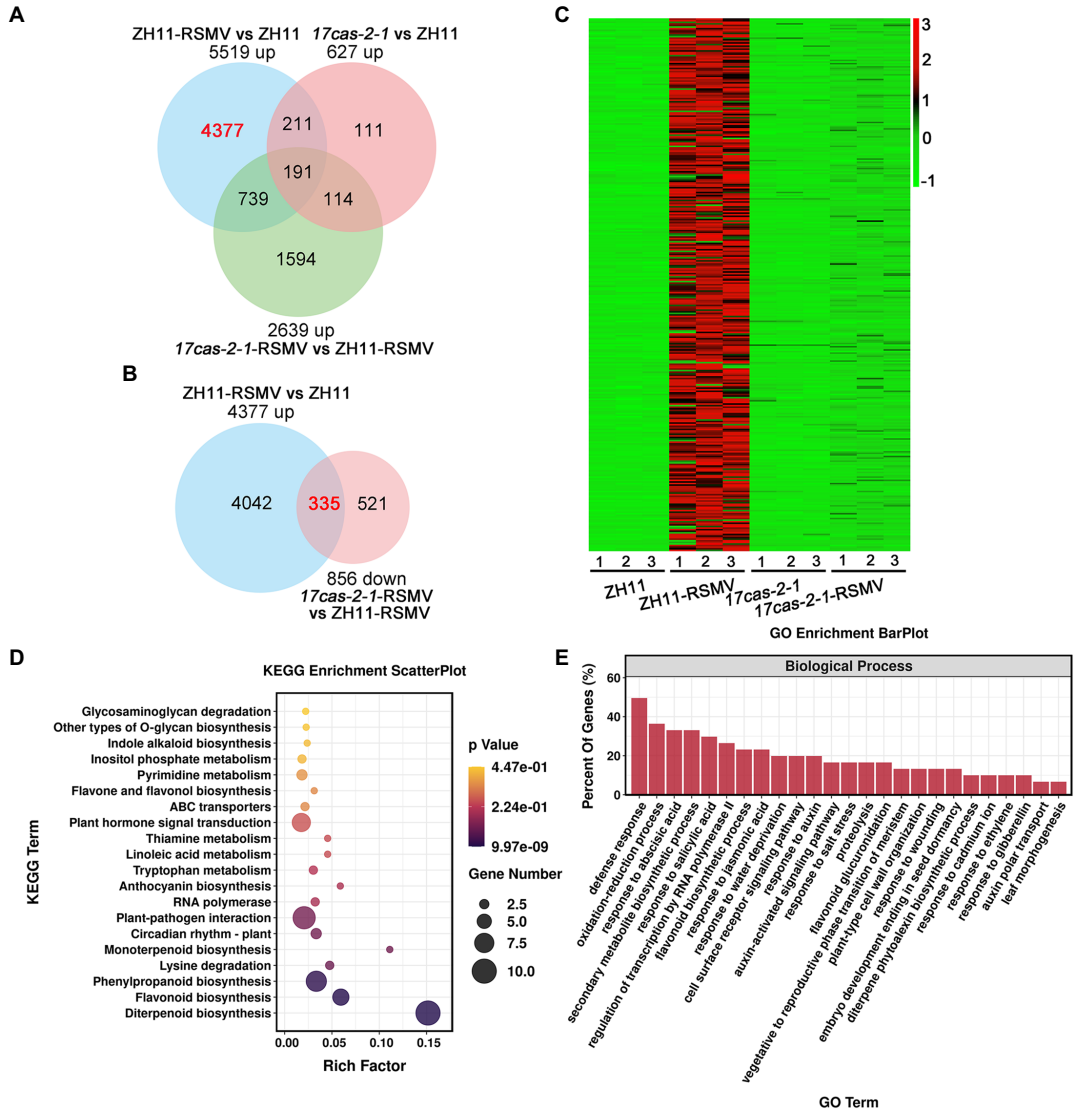
Analysis of the differentially expressed genes (DEGs) of overlaps of 4,377 specific up-regulated genes in ZH11-RSMV vs. ZH11 and 856 specific down-regulated genes in *17cas-2-1*-RSMV vs. ZH11-RSMV. **(A)** Venn diagrams showing overlaps of up-regulated differentially expressed genes in ZH11-RSMV vs. ZH11, *17cas-2-1*-RSMV vs. ZH11-RSMV and *17cas-2-1* vs. ZH11. **(B)** Venn diagrams showing overlaps of overlaps of 4,377 up-regulated genes in ZH11-RSMV vs. ZH11 and 856 down-regulated genes in *17cas-2-1*-RSMV vs. ZH11-RSMV. **(C)** Hierarchical clustering of 335 DEGs in **(B)** based on the log2 fold change in transcript levels in ZH11 and *OsARF17* mutant plants under mock or RSMV inoculation. The hierarchical clustering analysis was performed on the FPKM values of all tested genes. **(D)** KEGG pathway enrichment analyses of 335 DEGs labelled in red in **(B)**. “Rich factor” shows the ratio between the number of DEGs and the total genes in this pathway. **(E)** Gene ontology (GO) enrichment analysis of 335 DEGs labelled in red in **(B)**. The genes were summarized in biological process GO category. All differentially expressed genes were selected using cut-off *p*-value < 0.05 and fold-change > 2 compared with controls. Data were collected from three biological replicates, each containing a pool of three plants.

To learn more about the specific biological reactions of OsARF17 in rice resistance to RSMV infection and how it performed its function, we further analysis the DEGs in the comparisons ZH11-RSMV vs. ZH11 (4,377 up-regulated genes in Figure 4A) and *17cas-2-1*-RSMV vs. *17cas-2-1* (856 down-regulated genes in Figure 3A), and there was a high overlap of 335 genes between the two comparison groups (Figure 4B). According to the heat map analysis, we found that these genes were highly induced in RSMV-infected ZH11 plants compared with ZH11, but were not activated in *17cas-2-1* plants (Figure 4C).

The KEGG enrichment factor diagram, the related pathways involved in these 335 genes were as follows: Diterpenoid biosynthesis; Flavonoid biosynthesis; Phenylpropanoid biosynthesis; Lysine degradation; Monoterpenoid biosynthesis; Circadian rhythm-plant; Plant-pathogen interaction; RNA polymerase; Anthocyanin biosynthesis; Tryptophan metabolism; Linoleic acid metabolism; Thiamine metabolism; Plant hormone signal transduction; ABC transporters; Flavone and flavonol biosynthesis; Pyrimidine metabolism; Inositol phosphate metabolism; Indole alkaloid biosynthesis; Other types of O-glycan biosynthesis (Figure 4D). Consistent with the findings above, enrichment of KEGG pathway manifested that these overlapping genes were highly enriched in plant-pathogen interaction and plant hormone signal transduction. We also used GO analysis to classify the biological process of the 335 genes and found that they were mainly involved in the following biological processes: defense response; oxidation–reduction process; response to abscisic acid; secondary metabolite biosynthetic process; response to salicylic acid; regulation of transcription by RNA polymerase II; flavonoid biosynthetic process; response to jasmonic acid; cell surface receptor signaling pathway; response to auxin, auxin-activated signaling pathway and so on (Figure 4E). According to the results of the analysis, the majority of these 335 genes were involved in defense response and were linked to plant hormone responses including: abscisic acid; jasmonic acid; auxin and gibberellin. Previous research has shown that JA signaling cooperates with auxin, brassinosteroids, abscisic acid, and gibberellin pathways to activate rice antiviral immunity (He et al., 2017; Xie et al., 2018; Zhang et al., 2019; Li L. et al., 2022). Our results suggest that OsARF17-mediated auxin signaling may affect the interaction between the plant and RSMV by disrupting the interaction between different phytohormones and regulating defense gene expression in rice. Overall, the results of our analysis provide further insight into the physiological changes that occur in *OsARF17* mutant and ZH11 plants infected with RSMV and give us valuable information to better understand the role of OsARF17 in rice-virus interaction.

### Quantitative real-time PCR (RT-qPCR) verification on some defense gene expression profiles

**Figure 5:**
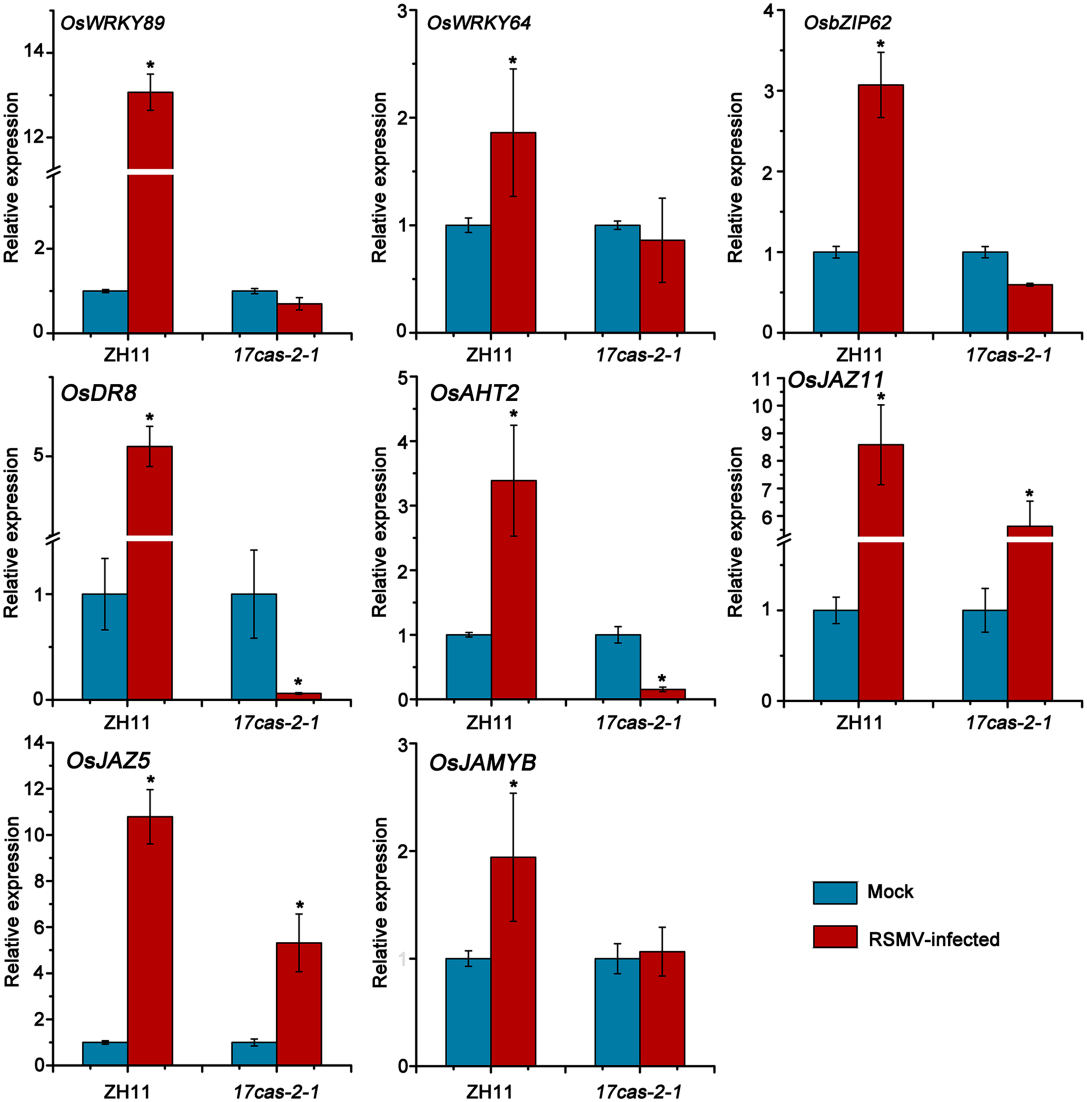
Results of RT-qPCR to verify the expression levels of 8 defense response-related genes in RSMV infected ZH11 and *OsARF17* mutant plants. ^*^ at the top of columns indicate significant differences (*p* < 0.05) based on Fisher’s least significant difference tests. UBQ5 was used as the internal reference gene.

The RNA-seq results indicated that a significant number of defense genes, such as *OsWRKY64* (Os12g02450), *OsWRKY14* (Os01g53040), *OsWRKY89* (Os11g02520), OsbZIP transcription factors of *OsbZIP70* (Os09g10840), *OsbZIP62* (Os07g48660), and gene related to disease resistance protein of genes related to disease resistance protein of *OsAHT2* (Os04g56910) and *OsDR8* (Os07g34570), were activated in ZH11 in response to RSMV infection, but were not activated in *17cas-2-1* plants (Table 1; Supplementary Tables S2–S6). Studies have reported that transcription factor OsWRKYs can influence the expression of PR genes, thereby activating the disease resistance response in rice (Wang et al., 2007; Zhang et al., 2018). In addition, the rice disease resistance-responsive genes, such as *OsDR8* and *OsAHT2*, played positive roles in rice defense against pathogens (Wang et al., 2006; Fang et al., 2022), therefore, these genes were selected and verified by RT-qPCR. Our previous results showed that the induction of JA in response to RBSDV was reduced in the auxin-related signaling mutants (Zhang et al., 2019). Our group has conducted extensive research on the JA signaling pathway and its related regulators (He et al., 2017). JA plays a positive role in antiviral defense in rice, while JAZ5, JAZ11 and JAMYB are key regulators of JA signaling, so we also validated them by RT-qPCR. As shown in Figure 5, the plant defense-related genes, such as WRKY transcription factors, *OsAHT2* and *OsDR8* were expressed notably more in ZH11 than in *OsARF17* mutant in response to RSMV infection. In addition, the expression of JA signalling-related genes (*OsJAZ5, OsJAZ11* and *OsJAMYB*) was markedly increased in RSMV-infected ZH11 plants compared to non-infected ZH11 plants. Interestingly, the expression of these genes in RSMV-infected *OsARF17* mutant was slightly increased compared to the mock-inoculated *17cas-2-1* plants (Figure 5). The results were consistent with the RNA sequencing data. These results suggested that JA pathway and defense-related genes activated by RSMV infection were suppressed when auxin transcription factor OsARF17 was deleted, further indicating that OsARF17 plays a positive regulatory role in the expression of these genes in rice antiviral immunity. Currently, we have only found that there may be regulatory relationships between OsARF17 and these defense-related genes. Further investigation using chip-sequencing, protein interaction, and the development and testing of various transgenic rice plants is needed to determine whether specific OsARF17 binds to the promoters of these genes and how it regulates them.

## Discussion

Our previous research had shown that RSMV M protein could interact with auxin response factor OsARF17, inhibiting the transcriptional activation of OsARF17, and benefiting the infection and replication of rice virus (Zhang H. et al., 2020). However, the role of OsARF17 in the antiviral mechanism remains unclear. In this study, *OsARF17* mutant (*17cas-2-1*) and control ZH11 plants in response to RSMV were used to as materials for a comparative transcriptome analysis. The results contribute to a more detailed understanding of virus-rice interactions at the molecular level.

We found that there were a large number of DEGs at the transcriptional level in the *OsARF17* mutant and ZH11 in response to RSMV, which was consistent with our previous study (He et al., 2017). Plants have evolved a variety of effective defense mechanisms against different pathogen attacks. Many defense genes were obviously up-regulated in the transcriptome data (Table 1). PRs, chitinases and MAPK were significantly induced in response to virus infection (He et al., 2017; Xie et al., 2018; Zhang et al., 2019, 2021). The *OsAHT2* gene was involved in APIP5-mediated defense response in rice, and overexpression of OsAHT2 in transgenic plants increased the content of phenolic amine metabolites and lignin, increased the transcriptional levels of defense-related genes *OsChitinase3* and *OsPR10*, and enhanced resistance to rice blast (Fang et al., 2022). *OsDR8*, a rice disease resistance-responsive gene, plays a positive role in rice defense against *Xanthomonas oryzae pv. oryzae* and *Magnaporthe grisea* (Wang et al., 2006). We found the expression levels of *OsAHT2* and *OsDR8* genes were significantly activated in ZH11 in response to RSMV infection (Figure 5). Additionally, many transcription factors, such as WRKY transcription factors, participated in the defense response and were upregulated (Table 1). WRKY transcription factors are plant-specific zinc finger type transcription regulators that contain a highly conserved 7 amino acid sequence at their N-terminus (Wu et al., 2005; Rushton et al., 2010, 2012). In *Arabidopsis thaliana*, a total of 74 WRKY members have been identified and 109 OsWRKY members in rice (Li et al., 2021). WRKY domain and its specific binding (T)(T) TGAC (C/T) sequence (also known as W-box) were more present in upstream regulatory regions of genes related to disease resistance, damage and aging and salicylic acid induction genes (Tian et al., 2006). In this study, the expression of *OsWRKY* genes, such as *OsWRKY14*, *OsWRKY64* and *OsWRKY89* were notably restrained in *OsARF17* mutants than that in ZH11 plants under RSMV infection (Figure 3C). Studies have reported that transcription factor OsWRKY14 affected the accumulation of 5-hydroxytryptamine to promote the expression of *PR* gene, thereby activating the disease resistance response in rice (Jin et al., 2015). Moreover, tryptophan (Trp) and its derived secondary metabolites (5-hydroxytryptamine, and 4-coumaryl serotonin) are important precursors for auxin biosynthesis (Höglund et al., 2019). OsWRKY14 may be play a key regulatory role in the biosynthesis of Trp and its secondary derivatives (Zhang et al., 2018). Therefore, the decreased expression of OsWRKY14 not only affect the expression of numerous resistance genes, including *PR* genes, but also affect the function of auxin signaling pathway. We speculated that downregulation of *OsWRKY* genes was due to deletion of OsARF17. Another member of the WRKY family, OsWRKY89, played a key role in biotic and abiotic stress response (Wang et al., 2007). The expression level of *OsWRKY89* was also inhibited in virus-infected mutants, suggesting that *OsWRKY* genes may be closely related to OsARF17 in rice-virus interaction. In future work, we aim to test whether OsARF17 protein can directly bind to the promoters of *OsWRKY* genes.

ARFs, as a class of transcriptional activators in the auxin signaling pathway, bind with the AuxRE element in the promoters to regulate the expression of auxin response genes (Waller et al., 2002; Guilfoyle and Hagen, 2007). ARFs play critical roles in plants normal growth and development (Ulmasov et al., 1999). For example, OsARF1 affects the crown root through modulating the expression of *OsCRL1* gene (Waller et al., 2002). OsARF12 influences the accumulation of iron in rice to regulate root elongation (Qi Y. et al., 2012). In addition, OsARF6 and OsARF17 through directly activating the expression of *ILA1* gene to control flag leaf angle (Qi L. et al., 2012). It is worth mentioning that 25 OsARFs in rice have diverse regulatory roles in antiviral immunity, such as OsARF12 and OsARF6 were sensitive to RDV infection, while OsARF11 mutants show enhanced resistance to RDV, this indicated that diverse OsARFs have difference functions in regulating rice antiviral ability (Qin et al., 2020). We found that OsARF17 played a positive regulatory role in rice resistance to RSMV infection (Figure 1). However, about the balance role of OsARF17 in plant growth and defense need to further research.

Plant hormones through synergistic or antagonistic interactions to regulate the defense response. In *Arabidopsis thaliana*, auxin defective mutants displayed more susceptible to necrotrophic pathogen than wild-type plants (Qi L. et al., 2012). In addition, auxin signaling plays an important role in plant defense against RBSDV (Zhang et al., 2019). JA signaling combined with auxin to activate rice antiviral immunity. Our recent results indicated that the activation of JA signaling was suppressed in auxin signaling mutants in response to viral infection (Zhang et al., 2019). In this study, KEGG and GO enrichment analysis revealed that the pathway term, “plant hormone signal transduction,” were markedly enriched (Figures 3C, 4D). Our results showed that the genes involved in JA signaling were obviously up-regulated in ZH11 plants under RSMV infection, while those in *OsARF17* mutants were slightly increased or no significant change (Figure 5). These observations imply that JA signaling genes normally induced by RSMV infection were inhibited when auxin signaling was damaged, suggesting that the activation of JA pathway was a part of OsARF17-mediated antiviral immunity of auxin. Among them, many genes associated with the plant hormone pathway have changed dramatically. For instance, *OsbZIP62/OsFD7* has transcriptional activation activity and participates in abscisic acid signaling pathway, which regulates the expression of stress-related genes such as *OsGL1*, *OsNAC10* and *DSM2,* and positively regulates drought and oxidative tolerance of rice.

Plants can accumulate large numbers of phytochemicals by biosynthesis and metabolic pathways, and virus invasion provokes many changes in plant metabolism pathways. Flavonoids, a class of important secondary metabolites produced in the metabolic pathway of phenylpropane in plants, not only participate in plant growth and development, but also regulate the response of plants to biotic and abiotic stress. KEGG analysis showed that “flavonoid biosynthesis” and “Diterpenoid biosynthesis” were significantly enriched (Figures 3C, 4D). Studies have shown that flavonoids can effectively inhibit the activities of PID, PIN and MDR-glycosidase, thus regulating auxin transport and the cascade amplification of auxin signals, affecting plant growth and development, and the immune system (Peer and Murphy, 2007). In addition, flavonoids secondary metabolites can induce the biosynthesis of jasmonate, and the synthesis of defense proteins, affecting the function of JA-mediated immune defense (Jiang et al., 2016; James et al., 2017; Wasternack and Strnad, 2019). Diterpenoid plays a key role in the biosynthesis of diterpenoid phytoalexins in rice (Yamamura et al., 2015; Zhao et al., 2019). We speculate that OsARF17 might be affect the biosynthesis and metabolites of flavonoids and diterpenoid, thus regulating rice resistance to virus. Jasmonyl-Ile conjugate hydrolase, jasmonoyl-isoleucine-12-hydroxylase and jasmonate O-methyltransferase activity are three enzymes with irreplaceable roles in the signal pathway of jasmonate synthesis and metabolism, the changes of contents will further interfere the antiviral signaling pathway of JA. These analysis results further show that the immune defense network mediated by OsARF17 may be involved a variety of plant hormones, metabolites such as flavonoids and diterpenoid, and plant antitoxins during the game struggle with viruses. Our experimental work further reveals the potential antiviral network mediated by OsARF17, providing a clue for understanding the cause of plant disease symptoms and antiviral immunity in rice.

## Conclusion

In our study, we used transcriptomic analysis to conduct comparative transcriptome analysis of *OsARF17* mutant (*17cas-2-1*) and wildtype ZH11 rice plants that were inoculated by RSMV. We identified 7,909 genes (6,908 up-regulated and 1,001 down-regulated) in *17cas-2-1* and 6,018 genes (5,518 up-regulated and 560 down-regulated) in ZH11 plants. The KEGG and GO enrichment analyses showed that these DEGs were mainly located in some metabolic and stress response categories, including plant-pathogen interaction, plant hormone signal transduction, diterpenoid biosynthesis, flavonoid biosynthesis and photosynthesis. We also verified the expression levels of defense response-related genes in RSMV infected by RT-qPCR assays. These results show that OsARF17-mediated auxin antiviral immunity may be involved a variety of plant hormones and metabolites, such as flavonoids and diterpenoid in rice. This study provides new insights into the in-depth research of the interaction mechanism between rice and viruses.

## Data availability statement

The data presented in the study are deposited in the NCBI repository, accession number PRJNA918805.

## Author contributions

QM, ZS, and HZ conceived the project and designed the experiments. QM, FW, and WS carried out the experiments with assistance from CH. QM, ZW, YL, and HZ analyzed and discussed the results. ZS and HZ wrote the manuscript. All authors contributed to the article and approved the submitted version.

## Funding

This work was funded by National Natural Science Foundation of China (32272555, 32270149, and 32001888), Young Elite Scientist Sponsorship Program by CAST (YESS20210121). This work was sponsored by the K. C. Wong Magna Fund in Ningbo University.

## Conflict of interest

The authors declare that the research was conducted in the absence of any commercial or financial relationships that could be construed as a potential conflict of interest.

## Publisher’s note

All claims expressed in this article are solely those of the authors and do not necessarily represent those of their affiliated organizations, or those of the publisher, the editors and the reviewers. Any product that may be evaluated in this article, or claim that may be made by its manufacturer, is not guaranteed or endorsed by the publisher.

## Footnotes

^1^ http://geneontology.org/

^2^ http://www.genome.jp/kegg

## Supplementary material

SUPPLEMENTARY FIGURE 1 The mutations of *17case* plants. **(A)** The mutants of OsARF17 with the nucleotide deletion were shown by red letters. *17cas-2-1* mutant harbored a deletion of TC and *17cas-5-2* with a deletion of TCACA, which generating a frameshift mutation leading to a premature stop codon. **(B)** Schematic diagram of the full-length and OsARF17 truncated. Three complete domains of OsARF17 in **(A)** were truncated. DBD: DNA-binding domain; MR: middle region; CTD: carboxy-terminal dimerization domain.
