# Supplementary figures and images for: Transcriptome analysis of auxin transcription factor OsARF17-mediated rice stripe mosaic virus response in rice
Source: Front Microbiol. 2023 Mar 9;14:1131212. doi: 10.3389/fmicb.2023.1131212 (PMC10033593; doi:10.3389/fmicb.2023.1131212)

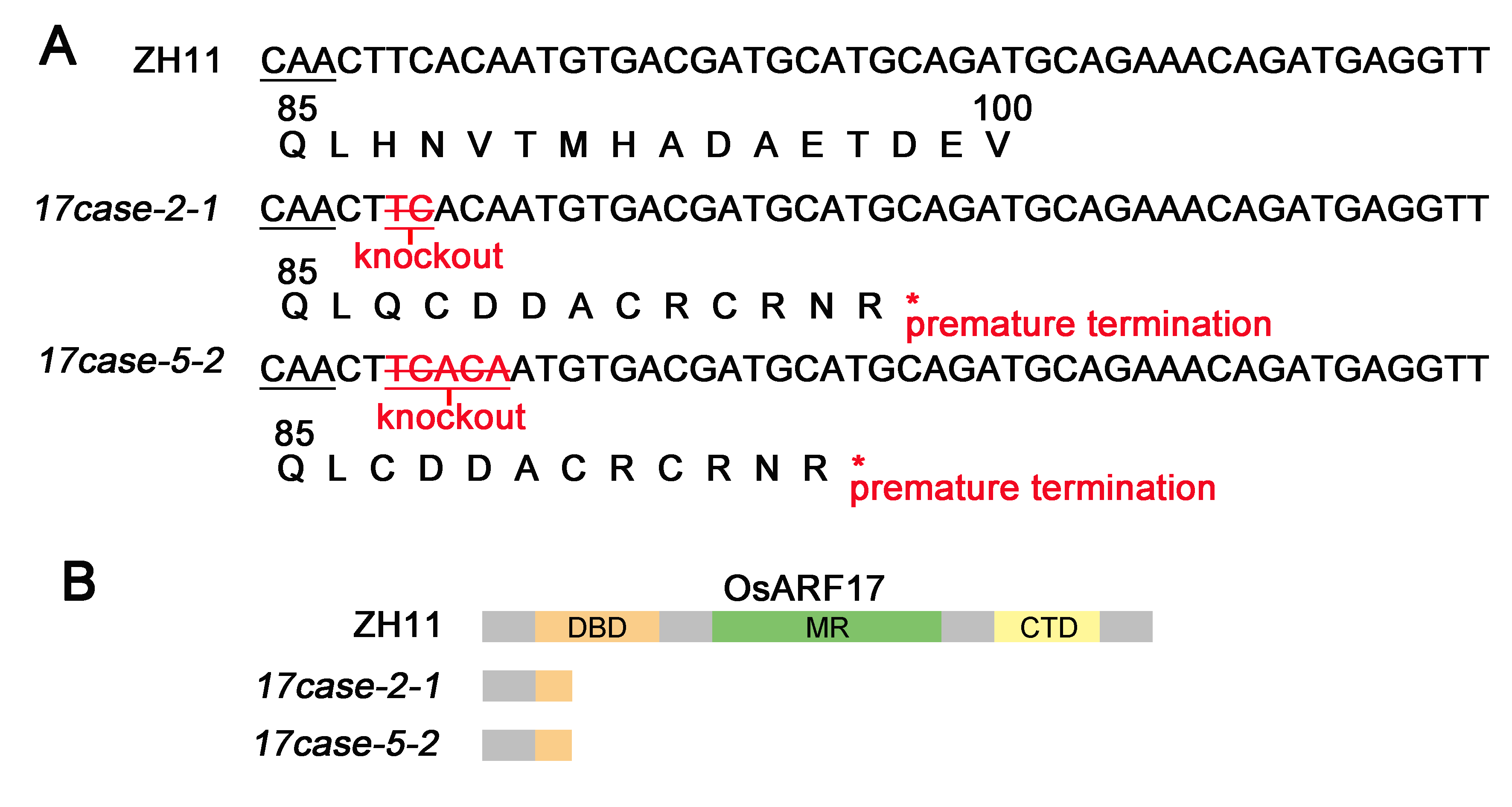

Supplement: SUPPLEMENTARY FIGURE 1 — The mutations of 17case plants. (A) The mutants of OsARF17 with the nucleotide deletion were shown by red letters. 17cas-2-1 mutant harbored a deletion of TC and 17cas-5-2 with a deletion of TCACA, which generating a frameshift mutation leading to a premature stop codon. (B) Schematic diagram of the full-length and OsARF17 truncated. Three complete domains of OsARF17 in (A) were truncated. DBD: DNA-binding domain; MR: middle region; CTD: carboxy-terminal dimerization domain. [file Image_1.TIF]
